# Detecting bursts in the EEG of very and extremely premature infants using a multi-feature approach

**DOI:** 10.1016/j.medengphy.2017.04.003

**Published:** 2017-07

**Authors:** John M. O’Toole, Geraldine B. Boylan, Rhodri O. Lloyd, Robert M. Goulding, Sampsa Vanhatalo, Nathan J. Stevenson

**Affiliations:** a Neonatal Brain Research Group, Irish Centre for Fetal and Neonatal Translational Research (INFANT), University College Cork, Ireland; b Department of Clinical Neurophysiology, Children’s Hospital, HUS Medical Imaging Center, University of Helsinki and Helsinki University Hospital, Helsinki, Finland

**Keywords:** Burst detection, Electroencephalography, Preterm infant, Feature extraction, Spectral analysis, Support vector machine, Inter-burst interval

## Abstract

*Aim:* To develop a method that segments preterm EEG into bursts and inter-bursts by extracting and combining multiple EEG features. *Methods:* Two EEG experts annotated bursts in individual EEG channels for 36 preterm infants with gestational age < 30 weeks. The feature set included spectral, amplitude, and frequency-weighted energy features. Using a consensus annotation, feature selection removed redundant features and a support vector machine combined features. Area under the receiver operator characteristic (AUC) and Cohen’s kappa (*κ*) evaluated performance within a cross-validation procedure. *Results:* The proposed channel-independent method improves AUC by 4–5% over existing methods (*p* < 0.001, n=36), with median (95% confidence interval) AUC of 0.989 (0.973–0.997) and sensitivity–specificity of 95.8–94.4%. Agreement rates between the detector and experts’ annotations, κ=0.72 (0.36–0.83) and κ=0.65 (0.32–0.81), are comparable to inter-rater agreement, κ=0.60 (0.21–0.74). *Conclusions:* Automating the visual identification of bursts in preterm EEG is achievable with a high level of accuracy. Multiple features, combined using a data-driven approach, improves on existing single-feature methods.

## Highlights

• Machine learning approach enables accurate detection of bursts in preterm EEG.
• Features of amplitude and spectral shape capture discriminating information.
• Improves reliability of estimates of inter-burst intervals.

## 1 Introduction

Preterm birth is the single largest risk factor for perinatal mortality and morbidity, accounting for over 1 million deaths every year [1]. The immature brain of the preterm infant is especially vulnerable and often the source of long-term health problems. The electroencephalogram (EEG) can help identify at-risk infants by providing continuous cot-side monitoring of brain activity in the neonatal intensive care unit (NICU). The EEG, however, requires interpretation by specialist staff which often makes it impractical to provide continuous reporting for all infants. Automated EEG analysis could overcome this limitation and provide the clinician with relevant information, in real time, to guide treatment during critical care.

Early preterm EEG exhibits an intermittent or discontinuous pattern (*tracé discontinu*) consisting of low-voltage activity, known as inter-bursts, followed by short-duration higher-voltage activity, known as bursts or spontaneous activity transients [2]. This pattern differs to the burst-suppression pattern found in the EEG of adults and full-term infants, a pattern associated with severe brain injury or coma [3]. In contrast, the discontinuous pattern is indicative of normal, healthy neurological development for the preterm infant. An important first stage for any automated analysis of preterm EEG is to distinguish between bursts and inter-bursts. Simple features of this bursting pattern, such as maximum inter-burst duration, relate to neurological development and are associated with neurological delay [4], [5], [6], [7]. Segmentation of the EEG into bursts and inter-bursts is an essential first-stage for more advanced automated analysis; for example to predict neurodevelopmental outcome [8], detect changes in sleep states [9], or assess changes in maturation [7].

Existing methods for detecting bursts in preterm EEG rely on either amplitude or frequency characteristics, or combinations of both [2], [6], [8], [10], [11], [12], [13], [14], [15], [16], [17], [18], [19]. Many of these methods, however, were not designed as stand-alone detection methods and have not been assessed with the gold standard, the EEG expert’s visual interpretation of the EEG [2], [8], [10], [11], [13], [16]. For those methods with performance validation metrics, the more promising methods employ frequency-weighted energy measures, which multiply amplitude and frequency to estimate energy [6], [17], [18], [19]. Yet the relative importance of amplitude and frequency features is unknown, and their optimal combination is as yet unexplored.

Here, we propose to assess multiple amplitude and frequency features separately and then combine these features in a classifier. This approach has been applied to detecting burst-suppression patterns in full-term EEG [20], [21]. Based on training from a large database of preterm EEG, machine learning algorithms can infer the best combination rules. We apply a feature selection procedure, that maximises relevancy and minimises redundancy, thus retaining only necessary features. Unlike existing methods, which either operate on 1 specific channel [17] or all channels simultaneously [6], [18], channels are processed independently as bursts can be focal or multi-focal and not always generalised across all channels. For example, in asynchronous activity bursts will not occur simultaneously across hemispheres [22]. For performance testing, feature sets and all parameters are estimated using strata of cross-validations to avoid overlap between training and testing data.

## 2 Methods

### 2.1 Acquiring and annotating the EEG

EEG data were collected from the NICU of the Cork University Maternity Hospital, Ireland, during the period 2009–2011. Data collection was approved by the Cork Research Ethics Committee of Cork Teaching Hospitals, Ireland. Informed and written parental consent was obtained before EEG recording.

EEG was recorded with the NicoletOne EEG system (CareFusion Co., San Diego, USA) using 11 electrodes according to the international 10–20 system of electrode configuration over the frontal, central, temporal, and occipital regions, a reference electrode at Fz, and a ground electrode behind the left ear. EEGs were recorded within 72 h of birth with a sampling frequency of 256 Hz. Infants with reported severe brain injuries, determined by cranial ultrasound scans within the first week of life, were not included.

Ten-minute segments with minimal artefact were selected from 36 EEG records (one segment per infant). These 10 min segments were, on average, 14 h post-birth (range: 3–41 h). Gestational age ranged from 23.4 weeks to 29.7 weeks with a mean of 27.4 weeks.

**Fig. 1:**
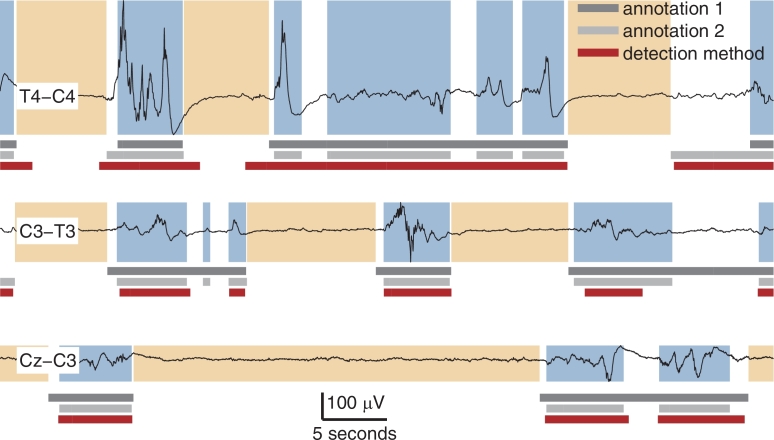
Annotations of bursts for 1-channel EEG recorded from 3 different preterm infants. Reviewers’ annotations (1 and 2) for bursts (labels) and inter-bursts (no labels) are used to generate a consensus annotation. Background shaded areas highlight this consensus annotation: blue for bursts and light brown for inter-bursts. Also included is the output from the proposed detection method. (For interpretation of the references to colour in this figure legend, the reader is referred to the web version of this article).

Two clinical physiologists (RO Lloyd and RM Goulding) annotated all EEG segments for bursts and inter-bursts. Bursts were defined as any preterm EEG activity not explicitly categorised as inter-bursts. Therefore the annotations included long-duration bursts (> 20 s) which some classification systems would label as continuous activity [4]. We chose not to distinguish between bursts and continuous activity because the difference between continuous and discontinuous activity is not clearly defined for infants with gestational age less than 32 weeks [4]. Example annotations are in Fig. 1.

EEG was analysed using the bipolar montage F4-C4, C4-O2, F3-C3, C3-O1, T4-C4, C4-Cz, Cz-C3, and C3-T3. EEG channels were annotated separately to develop a channel independent detector. As bursts do not always occur synchronously across all channels, a single channel was extracted for review to avoid annotation bias caused by the simultaneous display of multiple channels. One channel per infant was annotated and channel selection was alternated over all EEG records to avoid a channel bias. For example, F4-C4 was used for the first EEG, C4-O2 was used for the second, and so on. For all 36 EEGs, each channel was selected a median of 4.5 (range: 3–6) times.

Annotations differed between the two reviewers, as the example in Fig. 1 highlights. A consensus annotation, including only the burst or inter-burst periods where both reviewers agreed, was used for training and testing the classifier.

### 2.2 Feature set

**Fig. 2:**
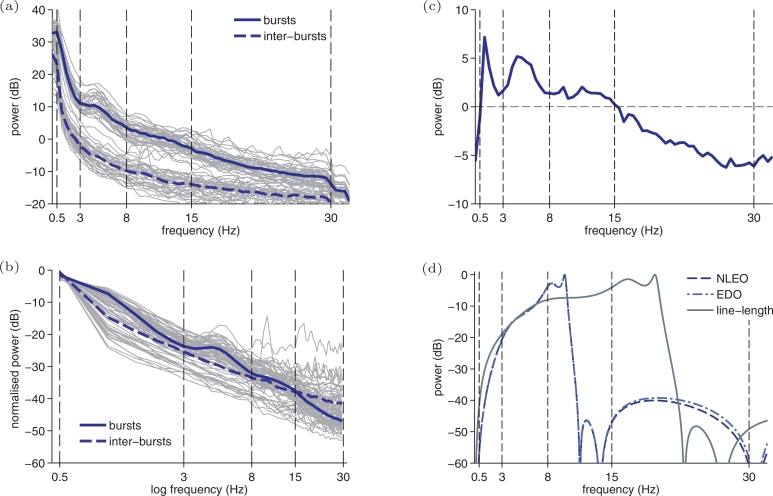
Spectral characteristics of bursts and inter-bursts with frequency responses of burst detection methods. Power-spectral density (PSD) estimates in (a) and (b) from 10 min EEG records of 36 preterm infants (grey thin lines) and median values (blue thick lines). PSDs are generated with Welch’s periodogram using a 2 s Hamming window. Normalised spectra in (b) is calculated by dividing by total spectral power in the 0–30 Hz region. Bursts-to-inter-bursts ratio in (c) is defined as the difference in median dB values in (b) between burst and inter-burst normalised spectra. Frequency responses in (d) for the nonlinear energy operator (NLEO), envelope–derivative operator (EDO), and line-length methods. These responses are plotted for comparison with the spectral characteristics in (a)–(c); responses are based on a single sinusoidal input and includes bandpass filtering (0.5–10 Hz for NLEO and EDO and 1–20 Hz for line-length). (For interpretation of the references to colour in this figure legend, the reader is referred to the web version of this article).

Fig. 2 highlights differences between bursts and inter-bursts. For example spectral power, across all frequencies, is greater for bursts comparative to inter-bursts [Fig. 2(a)]. Not surprising, considering amplitude plays a key role in many detection methods [2], [6], [8], [12], [17], [18], [19].

But also of interest are spectral characteristics independent of total power. Differences in relative spectral power is evident in the normalised spectra in Figs. 2(b) and the burst-to-inter-burst ratio (the difference in spectral power in dBs between the median burst and inter-burst spectra) in Fig. 2(c). Fig. 2(b) shows that the inter-bursts have an almost linear log–log frequency response compared with the more nonlinear response of the bursts. The following feature set aims to capture these differences in amplitude, relative spectral power, and spectral shape. These features are calculated within four frequency bands: band 1 (0.5–3 Hz), band 2 (3–8 Hz), band 3 (8–15 Hz), and band 4 (15–30 Hz) [2], [23].

##### 2.2.1 Amplitude features

Discrete EEG signal *x*(*n*) was bandpass filtered using a 5th-order Butterworth filter into the *i*th frequency band (i=1,2,3,4) to produce *x_i_*(*n*). These filters implement the forward–backwards procedure to produce a zero-phase filter. We calculated signal envelope *a_i_*(*n*) of *x_i_*(*n*) as

(1) $$a_{i}\left(n\right)={\left|z_{i}\left(n\right)\right|}^{2}={\left|x_{i}\left(n\right)+\text{j}H\left[x_{i}\left(n\right)\right]\right|}^{2}$$

where *z_i_*(*n*) is the analytic associate of *x_i_*(*n*); H represents the Hilbert transform and j represents the imaginary unit of the complex-valued *z_i_*(*n*).

##### 2.2.2 Spectral features

Multiple features are used to quantify spectral characteristics. Relative spectral power for the *i*th band is estimated as

(2) $$P_{i}=\frac{\sum_{k\in i}{\left|X(k)\right|}^{2}}{P_{\text{total}}}$$

where *X*(*k*) is the discrete Fourier transform (DFT) of length-*N x*(*n*), *P*_total_ is the total spectral power over the 0.5–30 Hz range, and notation ∑_*k* ∈ *i*_ represents summation over the *i*th frequency band.

To quantify spectral shape, we fit the line

(3) $$\hat{Y}\left(k\right)=c_{1}+c_{2}k$$

to the log–log spectrum *Y*(*k*) and then use slope *c*_2_ and measure-of-fit *r*^2^, defined as

(4) $$r_{i}^{2}=1-\frac{\sum_{k\in i}{\left[Y\left(k\right)-\hat{Y}\left(k\right)\right]}^{2}}{\sum_{k\in i}{\left[Y\left(k\right)-\frac{1}{N}\sum_{k\in i}Y\left(k\right)\right]}^{2}},$$

as features. This process has some similarity to a multifractal approach [24] but differs in the EEG frequency-band selection and summary measures.

Mean frequency is calculated using the periodic-mean frequency estimate,

(5) $$M_{i}=\frac{f_{s}}{4\pi }\left\{\arg\left[\sum_{k=0}^{N/2-1}|X_{i}\left(k\right){|}^{2}{\text{e}}^{j2\pi k/N}\right]\;\;\mathrm{mod}\;2\pi \;\right\}$$

with mod 2*π* representing the modulus function in 2*π, f_s_* the sampling frequency, and *X_i_*(*k*) is the DFT of *x_i_*(*n*). Instantaneous frequency is calculated using the central-finite difference estimate,

(6) $$f_{i}\left(n\right)=\frac{f_{s}}{4\pi }\left\{\left[\phi_{i}\left(n+1\right)-\phi_{i}\left(n-1\right)\right]\;\mathrm{mod}\;2\pi \right\}$$

with phase function $\phi_{i}\left(n\right)=\arg\left[z_{i}\left(n\right)\right],$ where *z_i_*(*n*) is the analytic signal described in (1).

We also include fractal dimension because of its association with spectral shape [25]. The Higuchi method first estimates curve length for scale value *k* as

(7) $$L_{m}\left(k\right)=\frac{\left(N-1\right)}{\left\lfloor \left(N-m\right)/k\right\rfloor k^{2}}\sum_{i=1}^{\left\lfloor (N-m)/k\right\rfloor }|x\left[m+ik\right]-x\left[m+\left(i-1\right)k\right]|$$

over m=1,2,…,k, using the entire frequency range 0.5–30 Hz for *x*(*n*). Curve length *L*(*k*), at scale *k*, is then computed as the mean value of *L_m_*(*k*) over all *m* values. This process is iterated for different scale values *k*. If the process is self-similar and stationary then $L\left(k\right)\propto k^{-D},$ where *D* is the fractal dimension. The slope of a line fit to the points (log *k*, log *L*(*k*)) provides an estimate of −D [25].

##### 2.2.3 Frequency-weighted energy features

Recent detection methods apply features of frequency-weighted energy measures [6], [17], [18], [19]. These measures produce an instantaneous estimate of signal energy that is dependent on both amplitude and frequency [19]. Palmu et al. used the absolute value of the nonlinear energy operator (NLEO) with a moving average window, defined as [17], [23]

(8) $$\Theta =\sum_{n=1}^{N-1}|x\left(n-1\right)x\left(n-2\right)-x\left(n\right)x\left(n-3\right)|$$

and Koolen et al. used the line-length measure [18]

(9) $$l=\sum_{n=1}^{N-1}|x\left(n+1\right)-x\left(n\right)|.$$

Although line-length was presented as a measure of fractal dimension [18], it better fits the definition of a frequency-weighted energy measure. Relating line length in (9) to curve length in (7), l=L(1) where *L*(1) is the intercept point on the log *L*(*k*)–log *k* plot. Because the intercept is independent of the slope, line length has no apparent relation to fractal dimension.

Both NLEO and line-length measures are not included in the feature set. Instead, we use the envelope–derivative operator which has similar properties to the NLEO but is non-negative [19]. The operator for discrete signal *x*(*n*) is defined as [19],

(10) $$\begin{array}{c} \Gamma \left(n\right)=\frac{1}{4}\left[x^{2}\left(n+1\right)+x^{2}\left(n-1\right)+h^{2}\left(n+1\right)+h^{2}\left(n-1\right)\right]\\ +\frac{1}{2}\left[x(n+1)x(n-1)+h(n+1)h(n-1)\right] \end{array}$$

where the discrete Hilbert transform *h*(*n*) is defined as IDFT{−jsgn(N/2−k)sgn(k)X(k)}; IDFT represents the inverse DFT and sgn represents the sign function.

NLEO and line length methods are compared with the proposed detector and are implemented according to published specifications [17], [18]: EEG is bandpass filtered (0.5–10 Hz for NLEO and 1–20 Hz for line length) and a moving average filter is applied to the output of the operator (1.5 s for NLEO and 1 s for line length). The bandpass filtering uses a 1st-order Butterworth filter for the high-pass component and a 6th-order elliptic filter for the low-pass component [17]. The envelope-–derivative operator is implemented with the same NLEO specifications (0.5–10 Hz pre-processing filter and 1.5 moving-average post-processing filter).

Although nonlinear functions, we present the frequency response of a single sinusoidal input in Fig. 2(d) for these frequency-weighting energy methods. For this diagram, the methods are implemented without the post-processing moving-average filter. The NLEO and envelope–derivative operator are implemented according to O'Toole et al. [19]; for the line-length, only the forward-difference component of the method is implemented, as the frequency response for x(n+1)−x(n) is known but unclear for |x(n+1)−x(n)|. Each frequency response includes the previously described pre-processing filters and are normalised within the 0–30 Hz region.

##### 2.2.4 Short-time analysis of features

For all features, except the frequency-weighted energy measures, EEG is down-sampled to 64 Hz. For these exceptions (NLEO, line-length, and envelope–derivative operator) the higher sampling rate (256 Hz) is used instead because of the known sensitivity to sampling frequency [6], [19]. Once calculated, the feature itself is then down-sampled to 64 Hz to ensure uniformity of sampling across all features.

Features are estimated within a short-time window, shifted in time with a 75% overlap, as detailed in Table 1. Spectral features use a 2 s window to include low-frequency activity at 0.5 Hz; amplitude and fractal dimension features use a 1 s window to allow for faster non-stationary activity.

**Table 1:** Feature set of 26 features. The 4 frequency bands are 0.5–3, 3–8, 8–15, and 15–30 Hz.

Feature	Analysis	Frequency
(relevant equation)	window (s)	band
Envelope–derivative operator (10)	1	0.5–10 Hz
Fractal dimension (7)	1	0.5–30 Hz
Envelope^a^(1)	1	4 bands
Relative PSD power (2)	2	4 bands
Mean frequency (5)	2	4 bands
Instantaneous frequency^a^(6)	2	4 bands
log–log PSD: slope (3)	2	4 bands
log–log PSD: *r*^2^(4)	2	4 bands

a median value of the analysis window. Key: PSD, power spectral density.

Features with asymmetric or heavy-tailed distributions are transformed using the natural log. Log-transformed features include line-length, NLEO, envelope–derivative operator, envelope, and spectral-power features. All features are then normalised to *z*-scores.

### 2.3 Feature selection and classification

Feature selection was implementing using the maximum-relevance–minimum-redundancy (mRMR) approach [26]. This method includes both a filter and wrapper stage. The filter stage, which is independent of the classifier, uses mutual information to find a feature subset that maximises relevance and minimises redundancy. The wrapper stage uses backwards elimination to rank feature subsets based on classifier performance. The reduced feature set from the filter stage allows implementation of the more sophisticated backwards-elimination procedure with a realisable computational load.

Next, features were combined using a support vector machine (SVM). We selected an SVM because of its successful application in other newborn EEG methods [21], [27]. SVMs can use different kernels to generate different decision boundaries [28]. In initial testing we found no significant improvement for the radial basis function over the linear kernel and thus implemented the linear kernel. The linear-kernel SVM can be expressed as the linear regression equation

(11) $$D\left[x\left(n\right)\right]=\sum_{p=0}^{K-1}w_{p}x_{p}\left(n\right)+b$$

where $w_{p}=\sum_{q=0}^{N-1}\alpha_{q}x_{p}\left(q\right)$ for *K* features $x\left(n\right)=\{x_{1}\left(n\right),x_{2}\left(n\right),\cdots ,x_{K}\left(n\right)\}$. For training data y(n)=±1, with 1 for bursts and −1 for inter-bursts, the algorithm estimates the parameters *b* and *α_q_*; the support vectors are the set **x**(*q*) for which *α_q_* ≠ 0. [28].

To produce a binary output indicating either bursts or inter-bursts, a threshold is applied to the discriminating function *D*[**x**(*n*)] in (11). We implement both the static threshold $T_{\text{thres}}=0$ and the infant-dependent (adaptive) threshold $T_{\text{thres}}=\text{mean}\left\{D\left[x\left(n\right)\right]\right\}$ [6], [18].

We set lower limits on the duration of bursts and inter-bursts to remove short-duration segments. These limits are estimated from the reviewers’ annotations by selecting the 2.5th percentile of burst (and inter-burst) duration.

### 2.4 Analysis of detection performance

**Fig. 3:**
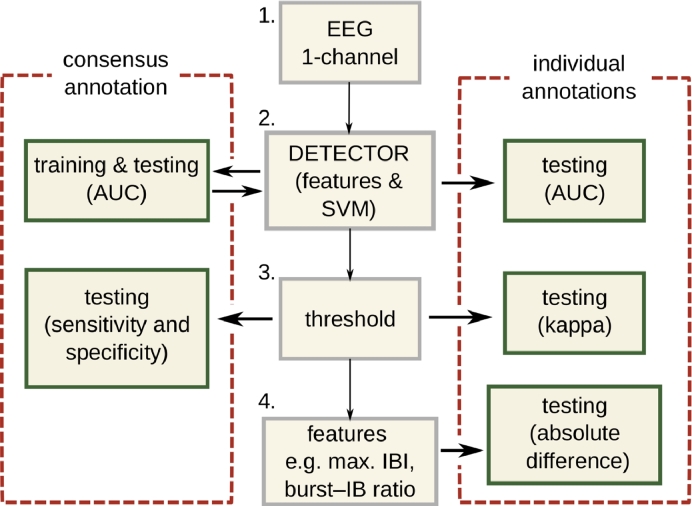
Training and testing for the burst detector. Consensus annotation (left) is derived from the two individual annotations of the human experts (right). The detector generates features from the EEG and combines them in the SVM (boxes 1–2). A threshold (box 3) then converts the continuous SVM output to a binary output that indicates bursts or inter-bursts. As part of the testing stage, features of the temporal distribution of the bursts, for example maximum IBIs, are estimated from this binary output (box 4) and compared with features derived from the human experts’ annotations. The detector is developed using the consensus annotation and tested using multiple metrics: AUC, sensitivity and specificity, Cohen’s kappa, and absolute difference between features of the burst annotations. Key: SVM, support vector machine; AUC, area under the receiver operator characteristic; IB, inter-burst; IBI, inter-burst interval.

The detector is developed using the consensus annotation; for testing, both consensus and individual annotations were used. Fig. 3 gives an overview of this process.

Both the individual features and the detector are assessed using the area under the receiver operator characteristic (AUC), generated from a time-based assessment on a sample-by-sample basis. AUC measures detection performance with values ranging from 0 to 1 where 0.5 representing random chance. Bursts were labelled as the signal-of-interest: true positives implies correct detection of bursts. For the individual features, an AUC was generated for each infant (n=36) and features were deemed statistically significant (*p* < 0.05) when the 95% CI of the AUC excluded 0.5.

Performance of the detector was assessed within a cross-validation procedure, thus avoiding over-fitting and reducing bias in testing error. Feature selection was implemented in a nested (inner) cross-validation for each outer training fold, as described in Ref. [29]. Both inner and outer cross-validation folds used a leave-one-out scheme, with testing on each left-out EEG record (one record per infant).

All parameters, including feature *z*-score parameters and SVM weights, were estimated within the training set and then applied to the testing set. Lower duration limits for bursts and inter-bursts were estimated from the reviewers’ annotations in the outer cross-validation. Features were generated from the EEG first. For feature selection and SVM training, only 1/500th of the training data (every 500th sample of the generated features) were used. This reduction in training data gave a good compromise between providing a representative distribution of values for both bursts and inter-bursts and computational efficiency during the training stage. For the testing stage, all available data was used.

AUC values for the NLEO and line-length methods [17], [18] were compared to the AUC (cross-validation testing results) for the proposed detector. In addition to the time-based assessment, we also include an event-based assessment for sensitivity–specificity. The event-based assessment quantifies detection performance independent of burst and inter-burst duration, defining a true positive when detecting more than 75% of the burst duration.

Inter-rater agreement between the two human experts is quantified using Cohen’s kappa statistic (*κ*) with the two annotations (Fig. 3). Bias and prevalence terms are reported with the *κ* statistic to better estimate agreement: prevalence quantifies the difference in the proportion of bursts to inter-bursts and bias quantifies the difference in the proportion of agreed bursts and inter-bursts. To assess the detector’s performance relative to inter-rater agreement, the detector is compared to each annotation separately using *κ*.

Three measures are calculated on the detector’s binary output: median inter-burst interval; maximum inter-burst interval; and burst-to-inter-burst ratio, the percentage of time the EEG is annotated as a burst per EEG record. These features represent important summary measures of preterm EEG as markers of normal maturation [4], [5], [6], [7], [9], [23]. These three features were also calculated using the reviewer’s annotation; absolute differences were calculated between the two annotations and the detection method, as indicated in Fig. 3.

Pair-wise comparisons use the Wilcoxon signed-rank test and include the median difference with a 95% CI. CIs are generated using the bootstrap method with 1000 iterations. *P*-values are reported with sample size *n*; in most instances n=36, the number of EEG records and infants in the study. When comparing the proposed detector to existing methods, we require *p* < 0.05 and at least a 1% improvement in performance to link statistical significance to engineering significance.

Finally, the detector was trained on all EEG records to generate a prototype burst detector suitable for validation on independent data. Matlab and Octave code for this detector (version 0.1.1) is provided in the Supplementary Material and updates are available at https://github.com/otoolej/burst_detector.

## 3 Results

**Fig. 4:**
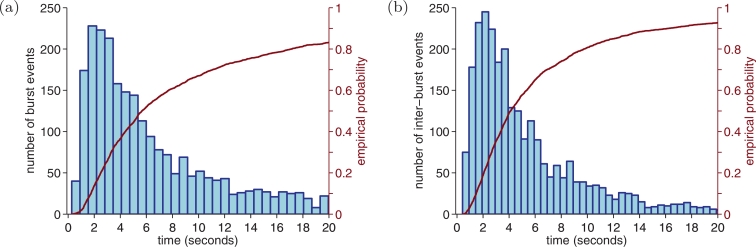
Distribution of the duration of (a) bursts and (b) inter-bursts periods using reviewers’ annotations. Individual annotations from the two reviewers are concatenated over all infants (n=36). Bursts are defined as valid EEG activity (non-artefacts) not categorised as inter-bursts. Plots limit maximum duration to 20 seconds although distributions do extend beyond this limit.

Fig. 4 shows the distribution of burst and inter-burst periods. Median (95% CI) burst duration is 5.7 (1.1–73.7) s, inter-burst duration is 4.1 (0.9–36.9) s, and burst-to-inter-burst ratio of 51% (32–86%). Lower-duration limits (2.5th percentile), over the cross-validation folds, had a median (95% CI) burst duration of 1.13 (1.12–1.17) s and inter-burst duration of 0.85 (0.84–0.88) s. The consensus annotation comprised of 77.5% of the total annotation.

**Fig. 5:**
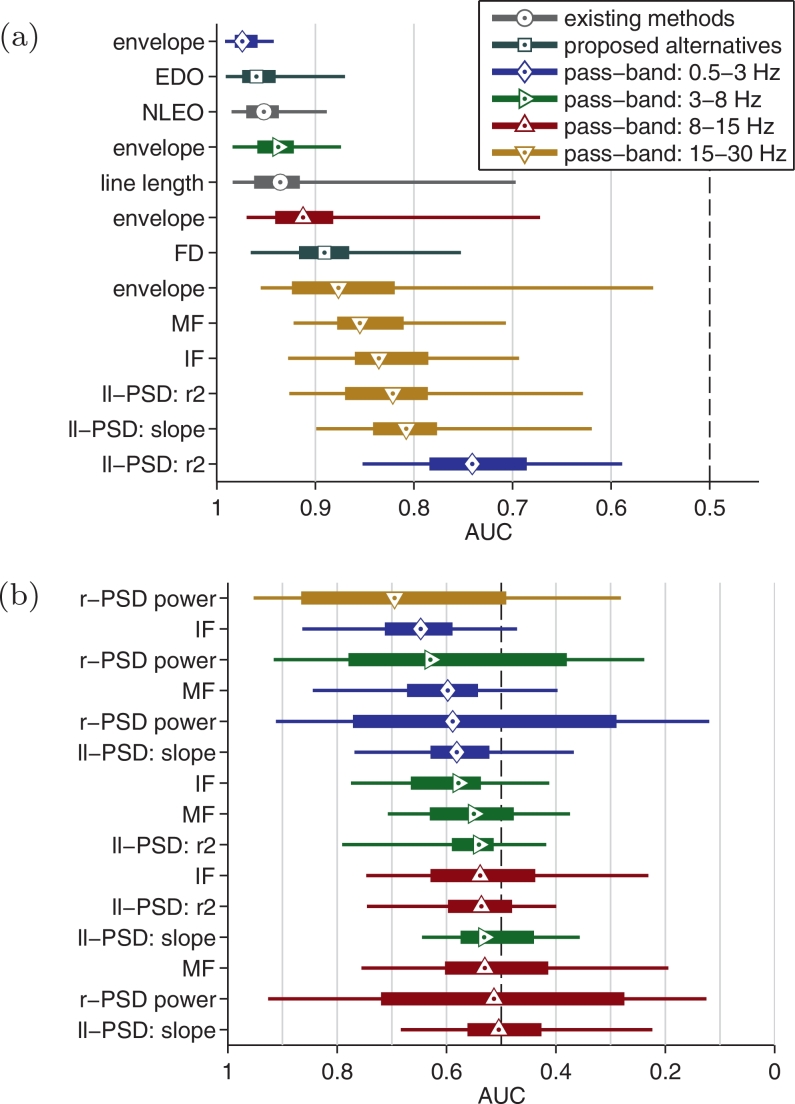
Detection performance for all 26 features of the feature set and 2 existing features, the NLEO and line-length. Features in (a) reach statistical significance (*p* < 0.05) as the 95% confidence interval excludes 0.5, whereas features in (b) fail to reach significance. Dots represent median values, thick lines represent inter-quartile range, and thin lines represent the 95th percentiles. Key: EDO, envelope–derivative operator; NLEO, non-linear energy operator; FD, fractal dimension; MF, mean frequency; IF, instantaneous frequency; ll-PSD, log–log power spectral density (PSD); r-PSD, relative PSD; AUC, area under the receiver operator characteristic.

Fig. 5 ranks detection performance for the 26 features in the feature set (Table 1); NLEO and line length methods are included for comparison. Less than one-half (11/26) of the features had significant detection performance. The 0.5–3 Hz envelope feature, with median (IQR) AUC of 0.974 (0.959–0.982), outperformed the NLEO (0.952, IQR: 0.937–0.970) and line-length (0.936, IQR: 0.916–0.962) features. The three frequency-weighted energy measures produced similar results, although the envelope–derivative operator, ranked second with median (IQR) AUC of 0.960 (0.940–0.974), had a slightly higher AUC (1–2%) than the NLEO and line-length AUCs.

Selected feature sets from the mRMR procedure over the cross-validation folds included a median of 9 (range: 8–12) features. Table 2 lists the selected features with frequency of occurrence.

**Table 2:** Frequency of selected features using the maximum-relevance–minimum-redundancy procedure over the 36 cross-validation folds. All features, except for the fractal dimension and EDO, are estimated over 4 frequency bands: 0.5–3 Hz (band 1), 3–8 Hz (band 2), 8–15 Hz (band 3), 15–30 Hz (band 4).

Frequency (%)	Feature	Frequency band
36 (100.0)	Fractal dimension	–
36 (100.0)	Envelope–derivative operator	–
36 (100.0)	Envelope	3
36 (100.0)	Envelope	4
35 (97.2)	Envelope	1
35 (97.2)	Relative PSD power	4
33 (91.7)	log–log PSD *r*^2^	1
29 (80.6)	Envelope	2
18 (50.0)	Mean frequency	4
12 (33.3)	Instantaneous frequency	4
6 (16.7)	log–log PSD slope	4
5 (13.9)	log–log PSD *r*^2^	4
5 (13.9)	instantaneous frequency	1
4 (11.1)	log–log PSD *r*^2^	2
4 (11.1)	log–log PSD slope	1
2 (5.6)	Instantaneous frequency	2
1 (2.8)	Relative PSD power	2
1 (2.8)	log–log PSD slope	2
1 (2.8)	log–log PSD *r*^2^	3

Key: PSD, power spectral density.

Table 3 shows a significant 4–5% improvement in AUC for the proposed detector over existing methods (*p* < 0.001; n=36). The detector also significantly improves over the best performing feature, the 0.5–3 Hz envelope feature, with a median (95% CI) increase in AUC of 1.55% (0.98–2.26%), *p* < 0.001 (n=36). Median (95% CI) sensitivity–specificity for the detector (using the static threshold) was 95.8% (77.3–99.7%) for sensitivity and 94.4% (66.7–99.5%) for specificity.

**Table 3:** Comparison of detection performance using the consensus annotations. % difference is between the proposed detector and other methods.

	AUC	% difference	*p*-value^a^
	median (95% CI)	median (95% CI)	
NLEO	0.952 (0.888, 0.988)	3.70 (2.40, 3.94)	< 0.001
line length	0.936 (0.694, 0.986)	5.25 (3.37, 6.04)	< 0.001
proposed	0.989 (0.973, 0.997)	–	–

a Wilcoxon signed-rank test. Key: AUC, area under the receiver operator characteristic; CI, confidence intervals; and NLEO, nonlinear energy operator.

**Fig. 6:**
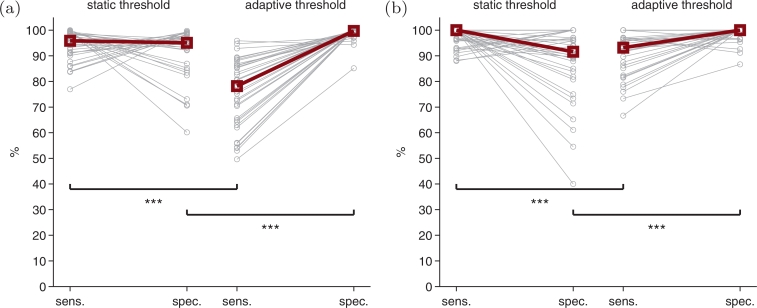
Detector using the static threshold, $T_{\text{thres}}=0,$ and adaptive threshold, $T_{\text{thres}}=\text{mean}\left\{D\left[x\left(n\right)\right]\right\},$ for (a) time-based assessment and (b) event-based assessment. Circles represent sensitivity (sens.) and specificity (spec.) for each infant, and squares represent median values. Statistical significance: *** *p* < 0.001, Wilcoxon signed-rank test.

Sensitivity–specificity using the time-based assessment for both static and adaptive thresholds is plotted in Fig. 6(a). Sensitivity is higher for the static threshold, with median (95% CI) difference between the static and adaptive thresholds of 17.9% (11.5–24.6%). But specificity is lower for the static threshold, with a difference between thresholds of −5.0% (−7.6% to −3.3%). Both differences are significant: *p* < 0.001, n=36. A similar picture emerges for the event-based assessment in Fig. 6(b): median (95% CI) difference between the static—adaptive thresholds is 1.2% (0.0–7.4%) for sensitivity and −8.7% (−11.5 to −3.4%) for specificity, with *p* < 0.001 (n=36) for both comparisons.

Table 4 shows inter-rater agreement together with the agreement between the detector and two reviewers, using AUC and *κ* as measures of agreement. Whereas the consensus annotation is used to train and test the detector, with results in Table 3, Table 4 presents testing results using the full individual annotations (see Fig. 3) and compares with inter-rater (reviewer) agreement. Fig. 1 shows examples of EEG segments comparing the two annotations to the detection method.

**Table 4:** Agreement between reviewers’ annotations (A1 and A2) and detection method.

	AUC	Cohen’s *κ*	bias, prevalence
	median (95% CI)	median (95% CI)	
detector vs. A1	0.844 (0.769, 0.916)	0.651 (0.316, 0.807)	0.08, 0.27
detector vs. A2	0.850 (0.649, 0.925)	0.721 (0.363, 0.831)	0.05, 0.16
A1 vs. A2^a^	0.815 (0.720, 0.879)	0.604 (0.213, 0.735)	0.15, 0.25

a Average AUC from A1 vs. A2 and A2 vs. A1. Key: CI, confidence intervals; AUC, area under the receiver operator characteristic.

**Fig. 7:**
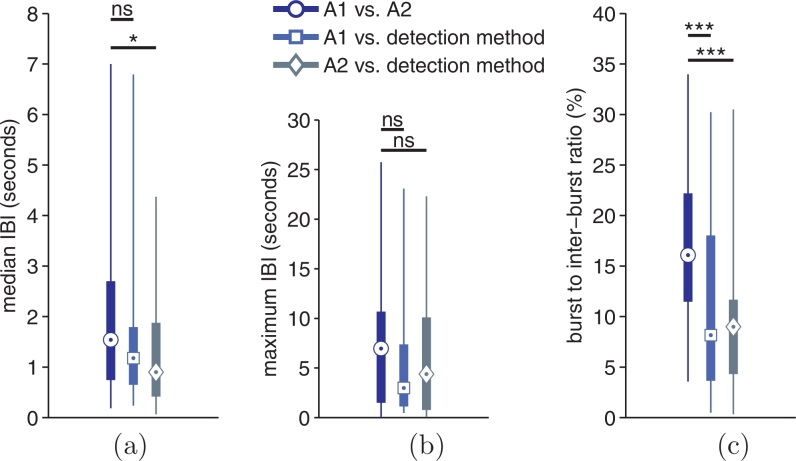
Differences in estimation of three features: (a) median duration of inter-burst interval (IBI), (b) maximum duration of IBI, and (c) ratio of bursts to inter-bursts. Plots show absolute differences for the 3 features between reviewers’ annotations (A1 and A2) and proposed detection method (using the static threshold). Pair-wise comparisons: either not significant (ns), * for *p* < 0.05, or *** for *p* < 0.001 using the Wilcoxon signed-rank test.

Fig. 7 shows differences in estimates of median and maximum inter-burst intervals and burst-to-inter-burst ratio, based on the annotations of the human experts and the proposed detector. Differences between detector and the two human experts is significantly lower than differences between human experts in 3 out of the 6 comparisons.

And lastly, we assessed processing speed for the proposed and existing methods. All methods were implement in Matlab (Release 2013a, The Mathworks Inc., Massachusetts, United States) on a desktop computer with a 2.8 GHz Intel Xeon processor and 8 GB of RAM. We used 2 h of EEG with 8-channels sampled at 256 Hz and processed each channel separately. The computational time was 64 s for the proposed method, 47 s for the NLEO method, and 1 s for the line-length method. Although slower than the single-feature methods, the proposed method is capable of processing EEG in real time.

## 4 Discussion

The proposed method outperforms existing methods for detecting bursts in preterm EEG, with improvements of approximately 4–5% in AUC (*p* < 0.001; n=36) over the frequency-weighted energy methods [17], [18]. Unlike these existing methods, the proposed method combines different features of amplitude and spectral content with a frequency-weighted energy measure. The cross-validation testing results—median AUC of 0.989 and sensitivity–specificity of 95.8–94.4%—show that the detector is capable of operating with a high-level of accuracy. The proposed method is also capable of analysing EEG in real time, with un-optimised code processing 2-h of 8-channel EEG in just over 1 min.

The 0.5–3 Hz envelope feature outperforms all frequency-weighted energy measures, with a difference in AUC of 2–4% (Fig. 5). An increase in low frequency amplitude is known to be associated with burst activity [2]. In contrast, the frequency-weighted energy measures suppress content within this band [Fig. 2(d)]. Although the frequency responses in Fig. 2(d) will differ for multi-component signals, their similarity for mono-components suggests that the pre-processing bandpass filters may be the most influential discriminating factor.

Most of the significant spectral features (5/7) are specific to the 15–30 Hz band (Fig. 5). And almost all spectral features (11/12) in frequency bands < 15 Hz performed poorly (*p* > 0.05). This suggests that frequency-weighted energy measures, which all operate < 20 Hz, rely heavily on amplitude and not on spectral characteristics. Yet the feature set always (Table 2) included the envelope–derivative operator, implying that there is value in including a feature which multiplies frequency by amplitude. In addition, both amplitude and frequency features were frequently (> 90%) included by the feature selection process (Table 2). Future work could develop features to further exploit spectral differences. For example, the burst-to-inter-burst spectral ratio in Fig. 2(c) could be applied in a spectral density correlator [30]. This type of matched filter correlates a received signal (EEG PSD) with a template (PSD estimate of bursts).

The two threshold methods, static and adaptive, produced similar results: better sensitivity with the static threshold and better specificity with the adaptive threshold. The static threshold may be a more robust approach however, as the adaptive threshold will fail in continuous or inactive EEG and will hinder a real-time implementation because of the required time-lag involved in threshold estimation.

Agreement between the detector and reviewer annotations was moderate (κ=0.65 and 0.72) with broad CIs, similar to agreement between the reviewers (κ=0.60). The seemingly high performance for the detector with the consensus annotation (AUC of 0.99, Table 3) drops to 0.84 and 0.85 when tested on the two annotations separately, reflecting the level of inter-rater agreement between human experts (Table 4). Our findings are consistent with known agreement rates: Palmu et al. reported rates of between 81% and 86% [23] and Murphy et al. reported rates of 71% with kappa values between 0.53 and 0.66 [6], although both studies included three, not two, annotations. This moderate inter-rater agreement highlights the inconsistencies in annotating bursts in preterm EEG and will limit the efficacy of any machine learning approach.

There is a clear advantage to an automated approach for the estimation of summary statistics of the burst annotation compared to visual interpretations (Fig. 7). Visual annotations will vary because of only moderate inter-rater agreement. The objectivity of the algorithm will decrease variability within these measures and therefore increase the reliability of preterm EEG analysis.

This study has several limitations. The proposed method was developed on EEG from infants with gestational ages ranging from 23 to 30 weeks, thus we are uncertain of how the method will perform for infants older than 30 weeks. The EEG data was largely artefact free, representative of a realistic sample of EEG used for visual analysis by clinical physiologists. For recordings with major artefacts, it may be necessary to include a pre-processing artefact detection system to assess the quality of the EEG [31], [32]. Although we have compared the method to existing detection methods, a fair comparison is difficult as methods were developed on different channel montages, with single channel or multi-channel implementations, and with different underlying definitions of bursts and inter-bursts [6], [17], [23]. Nonetheless, our results indicate that the multi-feature approach, with data-driven combination rules, better captures the complexity of the burst waveform compared to the single-feature approach. Although our method was developed on a larger EEG data set of preterm infants (n=36) compared to existing methods (n=18 and n=16 [17], [18]) the proposed method requires validation on a large, independent data set.

## 5 Conclusions

An important stage for the automated analysis of preterm EEG is to distinguish between bursts and inter bursts. We show that using a combination of features improves detection performance over existing methods. We also show that automated methods of detection improve the reliability of estimates of the median inter-burst interval and the burst-to-inter-burst ratio. Improving burst detection will improve downstream analysis of preterm EEG such as tracking maturation and predicting neurodevelopmental delay [7], [9].
